# Electronic Health Record Legal Settlements in the US Since the 2009 Health Information Technology for Economic and Clinical Health Act

**DOI:** 10.1001/jamahealthforum.2022.3872

**Published:** 2022-11-11

**Authors:** Nate C. Apathy, Jessica L. Howe, Seth A. Krevat, Aaron Zachary Hettinger, David W. Bates, David C. Classen, Raj M. Ratwani

**Affiliations:** 1 MedStar Health National Center for Human Factors in Healthcare, Washington, DC; 2 Brigham and Women’s Hospital, Boston, Massachusetts; 3 University of Utah School of Medicine, Salt Lake City

## Abstract

This cross-sectional study describes allegations of misconduct by electronic health record vendors.

## Introduction

The 2009 Health Information Technology for Economic and Clinical Health Act created incentives for clinicians and health care organizations to adopt electronic health records (EHRs).^1^ To receive incentive payments, clinicians and organizations are required to use EHRs that have been certified as meeting the capability, functionality, and security requirements adopted by the US Department of Health and Human Services (HHS).^2^ Some EHR vendors do not adhere to certain certification requirements yet their products are still certified and used.^3^ Postmarket surveillance of EHR products has revealed that some products do not meet certain certification requirements and may pose a risk to patient safety.^4^ Some certification violations have been investigated by the HHS Office of Inspector General and the US Department of Justice (DOJ). We analyzed publicly available settlement data to identify the nature of DOJ complaints related to EHR vendors and settlement amounts, and we calculated the number of clinicians using EHR products from vendors with alleged misconduct. For our purposes, we used the term *misconduct* to refer to only the alleged behavior by EHR vendors cited in the DOJ settlements.

## Methods

The DOJ’s online list of settlements was searched using the keywords *electronic health record* and *electronic medical record* to identify settlements with EHR vendors since 2009.^5^ The MedStar Health Research Institute Institutional Review Board deemed the study exempt from institutional review because all reports and data used were publicly available. We followed the (STROBE) reporting guideline.

Three of us (N.C.A., J.L.H., R.M.R.) independently reviewed each publicly available settlement summary to identify the EHR vendor, year and amount of settlement, and description of allegations. For each vendor named in a settlement, the number of unique clinicians attesting to using products from that vendor during the years of alleged misconduct (between 2011 and 2016) was identified from Meaningful Use Stage 1 and Stage 2 Public Use Files and the Office of the National Coordinator for Health Information Technology’s Certified Health Information Technology Products List.^6^ Data analysis was conducted in June 2022.

## Results

Six EHR vendors reached settlement agreements totaling $379.8 million (Table). Settlements for 5 of the 6 vendors involved alleged kickbacks, which are payments from the vendor to clinicians. Most kickbacks were related to product promotion, and 1 was related to influencing clinicians to prescribe opioids. Settlements for 4 of 6 vendors involved alleged misrepresentation of EHR capabilities to falsely certify their product. One vendor allegedly miscalculated rates of electronic record sharing, which were used in incentive program attestation. Based on available Centers for Medicare & Medicaid Services attestation data, the EHR products associated with these 6 settlements were used by 76 831 unique clinicians during the years of alleged misconduct.^6^

**Table.** US Department of Justice Settlements With EHR Vendors

EHR vendor	Settlement		Description of allegations	No. of clinicians attesting to meaningful use of an EHR product during the alleged period of misconduct
	Year	Amount in millions, $		
eClinicalWorks^a^	2017	155	Misrepresented EHR capabilities to certify a product (eg, hardcoding only the required drug orders for certification test) Paid kickbacks to certain customers for product promotion	35 451
Greenway Health LLC^b^	2019	57.25	Misrepresented EHR capabilities to certify a product (eg, hardcoding clinical vocabulary used in certification test) Paid kickbacks for product promotion Miscalculated interoperability measures reported to obtain meaningful use incentive payments	12 194
Practice Fusion Inc^c^	2020	145	Misrepresented EHR capabilities to certify a product (eg, EHR was unable to create standardized patient information export summaries) Solicited and received kickbacks from an opioid company in exchange for using the product to influence physician prescribing of opioids	6230
Viztek LLC^d^	2020	0.5	Misrepresented EHR capabilities to certify a product (eg, hardcoding software to pass certification testing)	39
athenahealth Inc^e^	2021	18.25	Paid unlawful kickbacks to generate sales of EHR products	22 526
CareCloud Health Inc^f^	2021	3.8	Paid unlawful kickbacks to generate sales of EHR products	391

Abbreviation: EHR, electronic health record.

^a^ https://www.justice.gov/opa/pr/electronic-health-records-vendor-pay-155-million-settle-false-claims-act-allegations

^b^ https://www.justice.gov/opa/pr/electronic-health-records-vendor-pay-5725-million-settle-false-claims-act-allegations

^c^ https://www.justice.gov/opa/pr/electronic-health-records-vendor-pay-145-million-resolve-criminal-and-civil-investigations-0

^d^ https://www.justice.gov/usao-nj/pr/new-jersey-electronic-health-records-company-pay-500000-resolve-false-claims-act

^e^ https://www.justice.gov/usao-ma/pr/athenahealth-agrees-pay-1825-million-resolve-allegations-it-paid-illegal-kickbacks

^f^ https://www.justice.gov/usao-sdfl/pr/miami-based-carecloud-health-inc-agrees-pay-38-million-resolve-allegations-it-paid

## Discussion

Most settlements included alleged kickbacks related to product promotion. This type of misconduct can contribute to a market in which usability, safety, and product effectiveness are not factors in product adoption, potentially leading to suboptimal products being widely used in patient care. The settlements involving alleged misrepresentation of EHR capabilities to certify a product are more concerning and may be associated with unsafe patient care if the product does not adhere to the usability and safety standards adopted by HHS. To improve the integrity of EHR certification, the certification process could be changed to ensure that EHR vendors are not aware of the specific tests that will be performed during certification. Furthermore, more frequent postmarket surveillance needs to be conducted to identify certification violations. Although these settlements involved only 6 vendors, at least 76 831 clinicians used their products, suggesting potentially far-reaching implications for patients.

This study has limitations. Some settlement details were not publicly available, and we limited the analysis to information made public by the DOJ and HHS. In these settlements, the EHR vendors did not admit wrongdoing and the reasons for the settlements were allegations by the DOJ.
